# The role of PI3K signaling pathway and its associated genes in papillary thyroid cancer

**DOI:** 10.1186/s43046-021-00068-2

**Published:** 2021-05-17

**Authors:** Elham Amjad, Solmaz Asnaashari, Babak Sokouti

**Affiliations:** Biotechnology Research Center, Tabriz University of Medical Sciences, Tabriz, Iran

**Keywords:** Papillary thyroid cancer, Meta-analysis, GEO datasets, Signaling pathway, Biomarker

## Abstract

**Background:**

One of the well-differentiated types of thyroid cancer is papillary thyroid cancer (PTC), often developed by genetic mutations and radiation.

**Methods:**

In this study, the public NCBI-GEO database was systematically searched. The eligible datasets were the targets for biomarker discovery associated with PI3K signaling pathway.

**Results:**

Only two datasets were suitable and passed the inclusion criteria. The meta-analysis outcomes revealed eleven upregulation and thirteen downregulation genes differentially expressed between PTC and healthy tissues. Moreover, the outcomes for survival and disease-free rates for each gene were illustrated.

**Conclusions:**

The present research suggests a panel signature of 24 gene biomarkers in diagnosing the PTC.

## Background

Papillary thyroid cancer (PTC), as one of the most prevalent cancer types diagnosed in the thyroid gland, covers more than 85% of total thyroid cancers [1, 2]. According to the fact that most of the large followed up thyroid cancer studies, specifically PTC, are epidemically carried out in the US nations, there are very few data available for monitoring the incidence of PTC worldwide [2]. Moreover, the well-known Surveillance, Epidemiology, and End Results database (SEER13) showed that the 5-year relative survival rate for 2010-2016 in the USA was 98.3% through age-adjusted incident rate with an unchanged trend for mortality rate of 0.4-0.5% [3]. Additionally, the Korean literature report indicated that the recurrence rate of patients with PTC after surgical treatment was less than 15% which led to survival by about 65% without including either 5- or 10-year survival rates [4]. However, various aggressive descriptors determined for PTC disease from imaging techniques have proposed a weak prognosis [4]. A recent case study reported that even if the diagnosis of PTC is somewhat tricky, combining a set of imaging techniques and fine-needle aspiration would make the road simplifying the PTC diagnosis [5].

The recent advancements in medical sciences have performed well by using microarray technology in narrowing down the list of diseases for diagnosis and prognosis procedures [6, 7], and on the other hand, the advances in technology will generate the Big data. In this regard, studying molecular and cellular functions of the particular diseases, e.g., the PTC, at genome-wide levels needs to be of priority. In other words, to differentiate between healthy and unhealthy tissues, the discovery of robust biomarkers is essential, either through experimental and clinical studies or computational biology approaches on Omics datasets [8]. There are several studies on investigating the molecular and physiological mechanisms of activation and inactivation of different signaling pathways having critical roles in the progress of PTC [9]. Since signaling pathways constitute hundreds of components, such as genes responsible for many cellular processes, their biological functions remain unclear and complicated [9].

Understanding the complex nature of PTC in terms of the involved signaling pathways urges studying vital roles of associated genes in the PI3K signaling pathway between PTC and healthy patients. For this purpose, the systematic search of the NCBI-GEO database retrieves the results of interest for further analysis and content screening based on inclusion and exclusion criteria. Finally, the genes of the selected GEO datasets will be the targets for identifying the differentially expressed genes among the datasets to be considered as robust biomarkers for PTC disease.

## Methods

### Identification of microarray datasets

The National Center for Biotechnology Information-Gene Expression Omnibus (NCBI-GEO) database (i.e., http://www.ncbi.nlm.nih.gov/geo) was the repository source for the systematic search of microarray datasets. For this search, a Boolean query was used, including the keywords “papillary thyroid cancer” or “PTC.” Furthermore, a thorough inspection on the search results was necessary for identification and inclusion of those GEO datasets in the analysis which satisfy the following criteria: (i) based on “expression profiling by array,” (ii) to be of [9606] organism, (iii) mRNA sample types, (iv) to have both PTC samples and healthy controls, and (v) extracted from the source of thyroid tumor. Notably, any platform types were of interest. Moreover, the excluded GEO datasets were those that not fulfilled the abovementioned inclusion criteria. Accordingly, the final selected GEO datasets with sufficient data were prone to perform a meta-analysis by including the associated genes of the PI3K signaling pathway.

### Associated genes for PI3K signaling pathway

The Kyoto Encyclopedia of Genes and Genomes (KEGG) database (i.e., https://www.genome.jp/kegg/pathway.html) was the resource to derive the involved genes in the PI3K signaling pathway (hsa04151) for Homo sapiens organism. Then, to carry out the meta-analysis approach among the GEO datasets, the associated genes of the PI3K signaling pathway were only considered for this purpose.

### Meta-analysis procedure

The ExAtlas free online tool for the meta-analysis of gene expression datasets possesses several main functions, such as the standard meta-analysis with fixed and random effects, z-score, and Fisher’s methods [10]. For this purpose, the input of the ExAtlas website included all GEO datasets meeting the inclusion criteria. However, among the whole gene symbols available in the GEO datasets, only those genes associated with the PI3K signaling pathway were selected for meta-analysis. The pre-processing stage of the input gene expression datasets comprised log2 transformation and quantile normalization applied to their corresponding intensity values as well as *t* test ANOVA analysis. After, a data quality check of the included samples was carried out based on the standard deviation criterion SD ≤ 0.3. Finally, the false discovery rate (FDR) and fold change parameters were 0.05 and 2, respectively, for the meta-analysis stage. Due to the heterogeneous nature of the gene expression datasets, the results for the random-effects model would be necessary.

### Analyses of survival and relapse-free rates

In survival analysis, various statistical methodologies analyzed the experimental data of interest in a defined period of follow-up time, usually, 200 months, in which death or relapse could happen carried out by plotting the Kaplan-Meier estimates [11, 12]. In this study, both overall survival (OS) and relapse-free survival (RFS) rates were considered on the Cancer Genome Atlas (TCGA)-THCA database professionally developed for thyroid carcinoma (*n* = 512) against control samples (*n* = 59). The Kaplan-Meier plots were obtainable using the GEPIA2 (http://gepia2.cancer-pku.cn/#index) web service [13]. In the KM-plot analysis, the *p*-values less than 0.05 were significant for input genes and the preset confidence interval for hazard ratio was 95%.

## Results

**Fig. 1:**
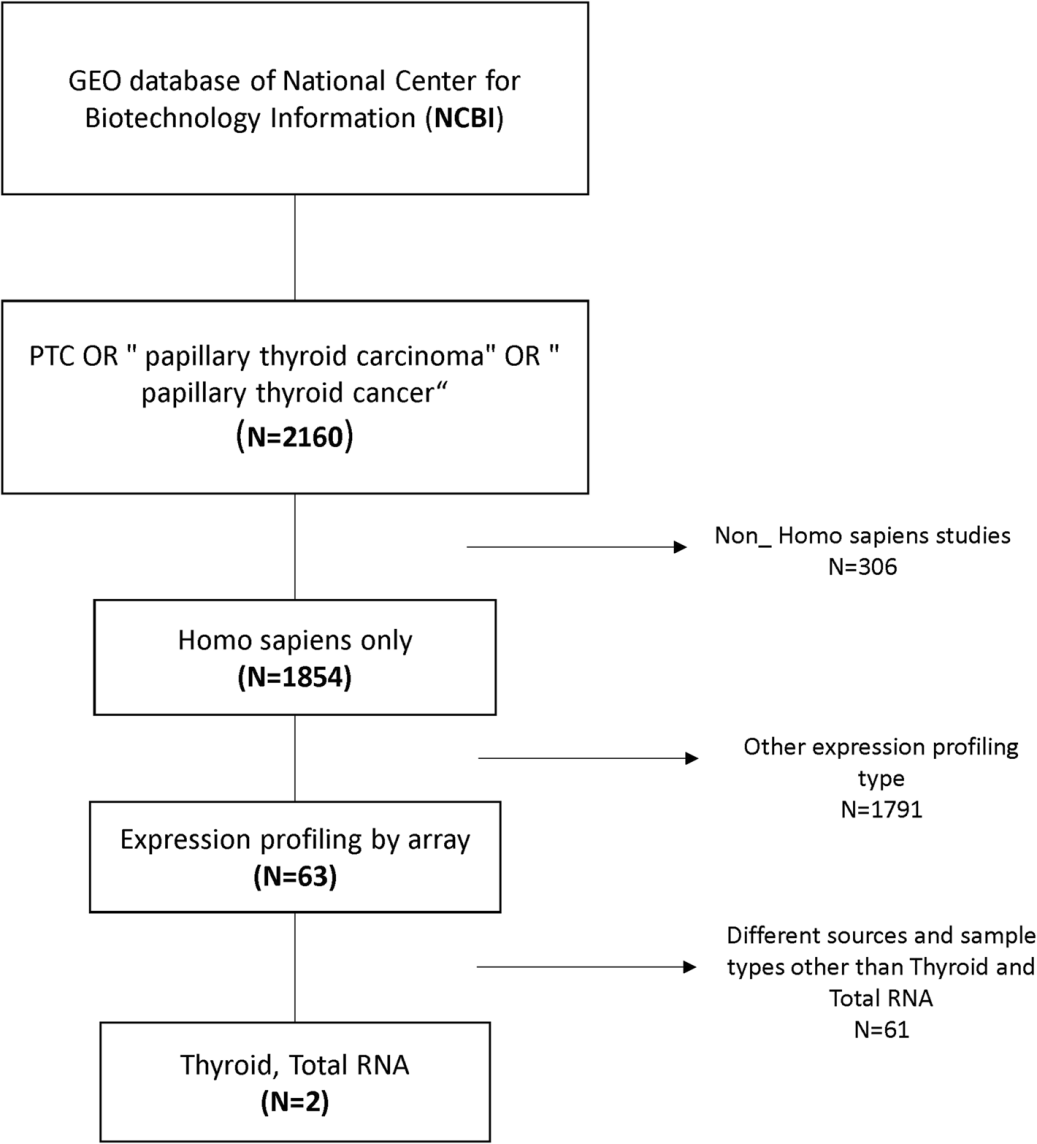
The flowchart for the data extraction from NCBI-GEO database

**Fig. 2:**
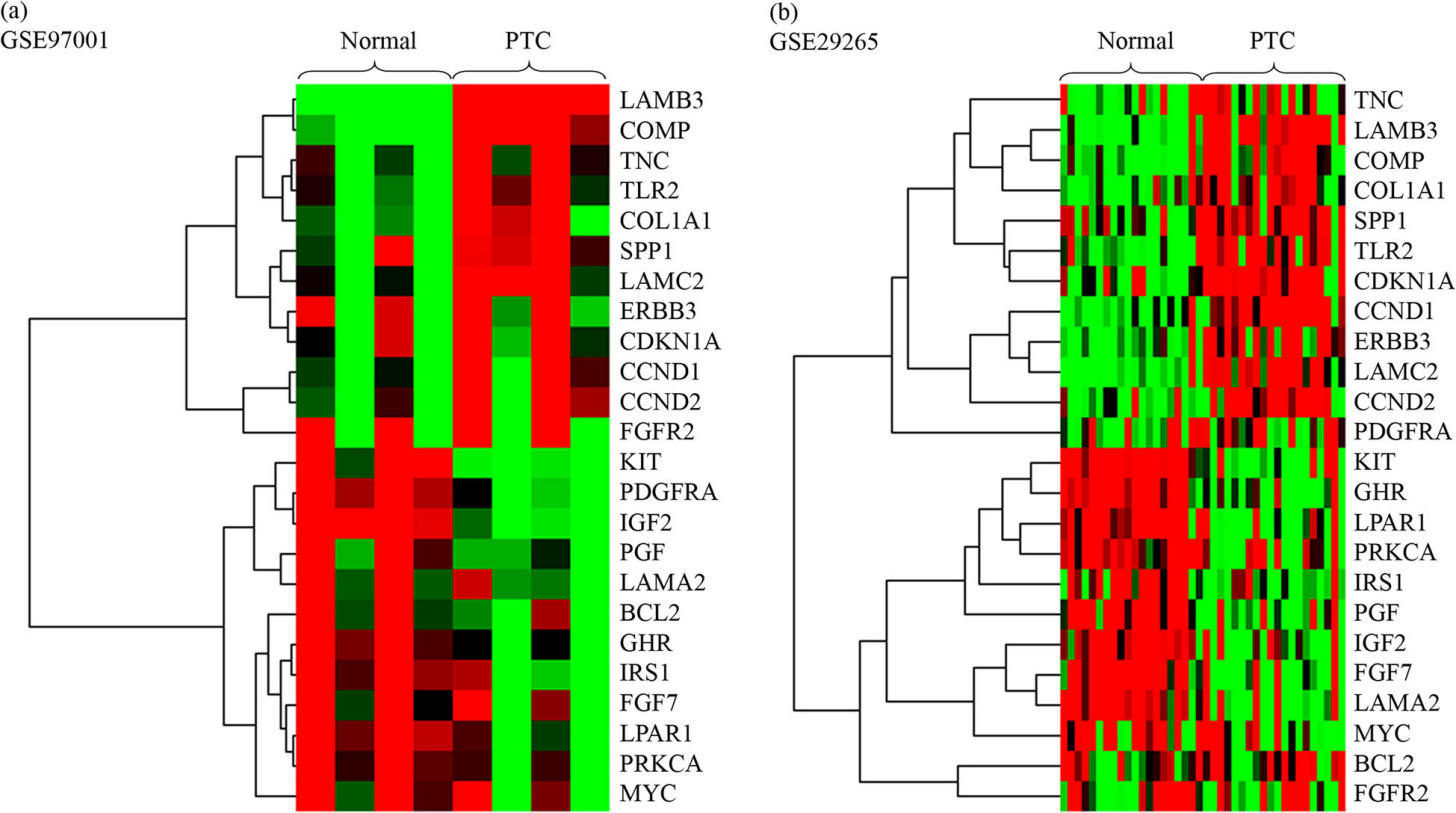
Cluster analysis of significant genes obtained from the meta-analysis approach for (**a**) GSE97001 and (**b**) GSE29265 datasets using Gene Cluster 3.0 and Java TreeView tools [14]. Upregulated genes with fold change combined values: LAMB3 (FC=23.836), COMP (FC=7.85), SPP1 (FC=3.605), TNC (FC=3.072), RBB3 (FC=2.842), CCND1 (FC=2.734), TLR2 (FC=2.591), CCND2 (FC=2.475), LAMC2 (FC=2.452), CDKN1A (FC=2.25), COL1A1 (FC=2.146); downregulated genes with fold change combined values: KIT (FC=9.05), PDGFRA (FC=4.126), IGF2 (FC=3.734), GHR (FC=3.626), BCL2 (FC=3.114), IRS1 (FC=3.038), LPAR1 (FC=2.67), FGF7 (FC=2.362), PGF (FC=2.31), FGFR2 (FC=2.245), PRKCA (FC=2.242), LAMA2 (FC=2.239), MYC (FC=2.025)

The overall results obtained from systematically screening of the NCBI-GEO database, where the data extraction procedure is depicted is Fig. 1, by taking in to account the inclusion and exclusion criteria showed that only two microarray datasets (i.e., GSE29265: 20 Normal and 20 PTC and GSE97001: 4 Normal and 4 PTC) were eligible for meta-analysis procedure. Three hundred fifty-four out of a total of 354 genes involved in the PI3K signaling pathway were selected from the two GEO datasets to identify the significant genes differentially expressed between the two tissue types. The ExAtlas website demonstrated that all of the samples included in the GEO datasets passed the initial quality control, which then was suitable for the meta-analysis process based on a random-effects model due to the existence of possible heterogeneity. The meta-analysis revealed twenty-four genes significantly expressed in terms of *p*-value and FDR parameter between healthy and PTC samples, among which the numbers of upregulated and downregulated genes were eleven and thirteen, respectively, as represented in Fig. 2.

**Fig. 3:**
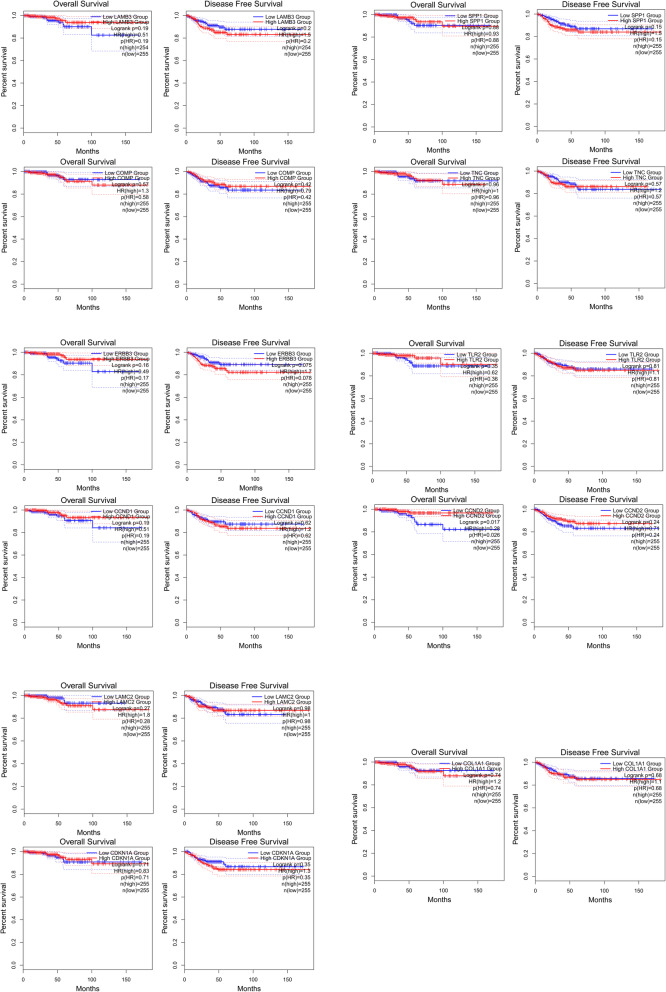
The survival analyses of identified upregulated genes in terms of OS and RFS

**Fig. 4:**
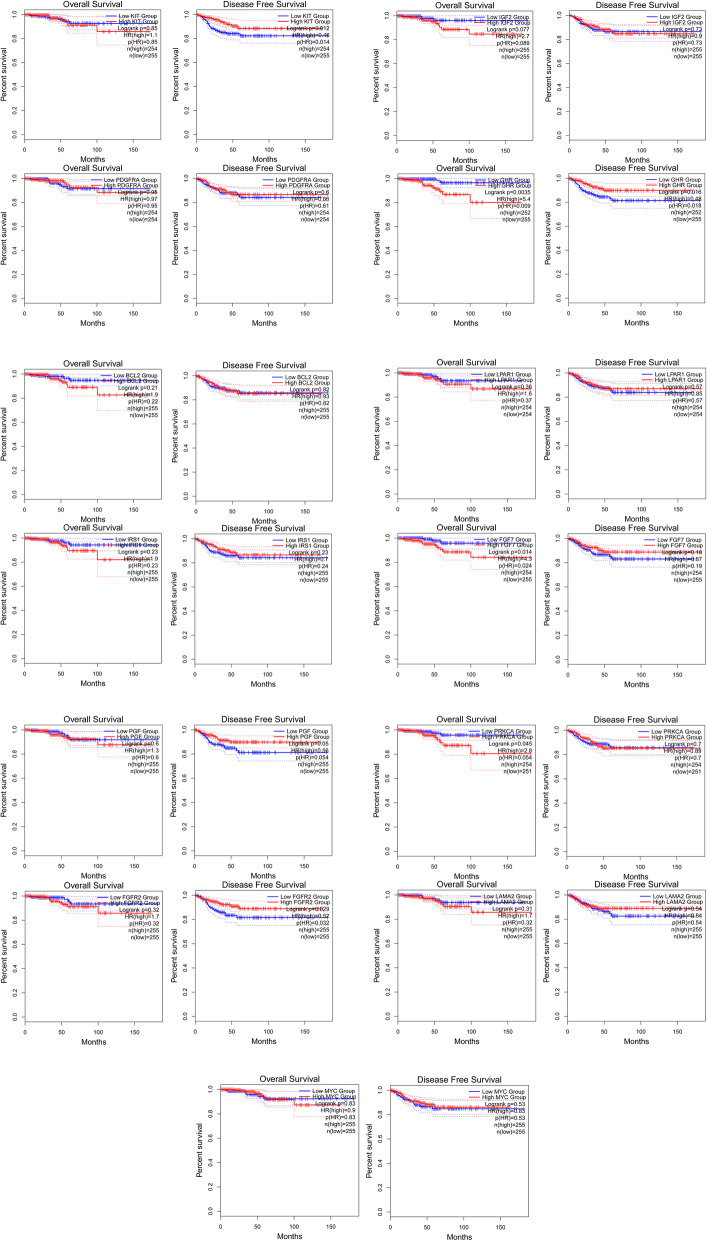
The survival analyses of identified downregulated genes in terms of OS and RFS

The overall survival (OS) and disease-free rates (RFS) for the identified upregulated and downregulated genes are illustrated in Figs. 3 and 4, respectively. Among upregulated genes, CCND2 was the only significant gene in terms of OS rate with p-value 0.017. Moreover, by considering the downregulated genes, three genes (i.e., GHR *p*-value=0.0035, FGF7 *p*-value=0.014, PRKCA *p*-value=0.045) were found to be significant in terms of OS rate; however, four genes (i.e., KIT *p*-value=0.012, GHR *p*-value=0.016, PGF *p*-value=0.05, FGFR2 *p*-value=0.029) were significant in terms of RFS rate.

## Discussion

Despite the excellent reports on the prognosis of the papillary thyroid carcinoma (PTC) as the predominant form of thyroid cancer, estimating the overall survival of PTC patients has still been remained unknown [15]. In the current research, a meta-analysis approach could demonstrate the significant differentially expressed genes between two GEO datasets meeting the inclusion criteria with FDR<0.05. Avoiding any possible inconsistency between the datasets is critical such that only the GEO datasets with the source of PTC tissues were eligible. In total, 24 genes (11 upregulated and 13 downregulated) were differentially expressed in PTC patients while being compared to the healthy samples with the statistical significance of FDR <0.05 and *p*-value <0.05 (Table 1).

**Table 1:** The list of 24 genes consistently expressed differentially between two GEO datasets associated with the PI3K signaling pathways in PTC patients seen in the literature

Gene symbol	Chromosome (source: https://www.genecards.org/)	Disease (source: https://www.genecards.org/)	References for associated genes and PTC
KIT	4q12	Gastrointestinal stromal tumors (GISTs), melanomas, lung cancer, and other tumor types	[16, 17]
PDGFRA	4q12	Idiopathic hypereosinophilic syndrome, somatic and familial gastrointestinal stromal tumors, and a variety of other cancers	[18, 19]
IGF2	11p15.5	One of the cancer-related genes	[20]
GHR	5p13.1-p12	Laron syndrome and growth hormone insensitivity, partial	[21]
BCL2	18q21.33	High grade B cell lymphoma with Myc and/or Bcl2 and/or Bcl6 rearrangement and follicular lymphoma 1	[22]
IRS1	2q36.3	Diabetes mellitus, noninsulin-dependent and rare diabetes mellitus type 2	[23]
LPAR1	9q31.3	Pulmonary fibrosis and spinal stenosis	[24]
FGF7	15q21.2	Mucositis and acanthoma	[25]
PGF	14q24.3	Placental insufficiency and twin-to-twin transfusion syndrome	[26]
FGFR2	10q26.13	Lung and breast cancers	[27]
PRKCA	17q24.2	Chordoid glioma and papillary glioneuronal tumors	[28]
LAMA2	6q22.33	Muscular dystrophy, congenital merosin-deficient, 1A and muscular dystrophy, limb-girdle, autosomal recessive 23	[29]
MYC	8q24.21	Burkitt lymphoma and high grade B cell lymphoma with Myc and/or Bcl2 and/or Bcl6 rearrangement	[30]
LAMB3	1q32.2	Epidermolysis bullosa, junctional, Herlitz type, and epidermolysis bullosa, junctional, non-Herlitz type	[31]
COMP	19p13.11	Pseudoachondroplasia and epiphyseal dysplasia, multiple, 1	[32]
SPP1	4q22.1	Pediatric systemic lupus erythematosus, and papillary cystadenocarcinoma	[33]
TNC	9q33.1	Deafness, autosomal dominant 56, and autosomal dominant non-syndromic sensorineural deafness type Dfna	[34]
ERBB3	12q13.2	Lethal congenital contracture syndrome 2 and erythroleukemia, familial	[35, 36]
CCND1	11q13.3	Von Hippel-Lindau syndrome and myeloma, multiple	[37]
TLR2	4q31.3	Leprosy 3 and colorectal cancer	[38]
CCND2	12p13.32	Megalencephaly-polymicrogyria-polydactyly-hydrocephalus syndrome 3	[39]
LAMC2	1q25.3	Epidermolysis bullosa, junctional, Herlitz type, and epidermolysis bullosa	[40]
CDKN1A	6p21.2	Multiple endocrine neoplasia, type I, and tongue cancer	[41]
COL1A1	17q21.33	Caffey disease and osteogenesis imperfecta, type I	[42, 43]

The above-listed genes in Table 1 were thoroughly inspected for their confirmation through various experimental studies considering the neoplastic thyroid disease. The expressions of associated genes in the PI3K signaling pathway were half downregulated and half upregulated. Taking in to account that some studies have reported on the target overexpressed gene whereas in the current study was determined as downregulated, and vice versa, the main reasons for this may generally originate from several points such as viral infections, patients clinical history, treatment status, the source of control samples, the age of patients as well as patients’ race to mention a few (e.g., upregulation and downregulation of has-mir-345 in pancreatic cancer [44, 45]). Moreover, in the current meta-analysis study, statistical stages, including data normalization, *t* test, and ANOVA tests were performed on GEO datasets to compare the PTC and healthy tissues in the same conditions. Due to the error-prone nature of the clinical and experimental trials, several biases (e.g., publication, laboratory, environmental, and user biases) may affect the reported outcomes by the researchers, and hence, the gene expression levels may not be comparable [46]. The determined genes (with FC>2) in the current meta-analysis study can be useful in identifying potent biomarkers for future drug design and discovery. Among the identified biomarkers associated with the PI3K signaling pathway, four of them with FC > 4 were LAMB3 (upregulated), COMP (upregulated), KIT (downregulated), and PDGFRA (downregulated). Various studies have also confirmed the vital role of the activation of the PI3K signaling pathway in the progression and development of PTC disease [47, 48]. As described in the “Results” section, seven genes were significant while considering the OS and RFS rates; however, this outcome will not decline the fact that the other remaining biomarkers have vital roles in the development of the PTC disease.

Consequently, in the current meta-analysis research, a total of twenty-four genes associated with the PI3K signaling pathway were identified and thoroughly screened and validated via the experimental literature studies that could propose a panel of potential biomarkers in PTC disease.

## Conclusion

The present study conducted on PTC GEO datasets revealed the significant role of the meta-analysis approach in determining the potential biomarkers for the disease. Eleven upregulated and thirteen downregulated genes were identified and validated through the literature investigations. By performing a meta-analysis study, one may conclude this type of analysis can fill the gaps between the computational and experimental studies; however, due to the existence of possible heterogeneities among the datasets, some of the differentially expressed genes may be missed that may urge novel algorithms to cover the shortcomings. The biomarker discovery is one of the hot topics in the field, which still needs more advancements in terms of technical, experimental, and computational designs to achieve more robust and reliable biomarkers, and hence, to provide its vital role in diagnosis, prognosis, and treatment of diseases.

## Data Availability

Not applicable

## Abbreviations

PTC: Papillary thyroid cancer
KEGG: Kyoto Encyclopedia of Genes and Genomes
mRNA: Messenger ribonucleic acid
NCBI: National Center for Biotechnology Information
GEO: Gene Expression Omnibus
PI3K: Phosphoinositide 3-kinases
ANOVA: Analysis of variance
TCGA: The Cancer Genome Atlas
RFS: Disease-free rates
OS: Overall survival
FDR: False discovery rate

## References

[1] Guo Z, Ge M, Chu Y-H, Asioli S, Lloyd RV. Recent advances in the classification of low-grade papillary-like thyroid neoplasms and aggressive papillary thyroid carcinomas: evolution of diagnostic criteria. Adv Anat Pathol. 2018;25(4):263–72. 10.1097/PAP.0000000000000198. 29762157 10.1097/PAP.0000000000000198

[2] Rossi ED, Pantanowitz L, Hornick JL. A worldwide journey of thyroid cancer incidence centred on tumour histology. Lancet Diab Endocrinol. 2021;9(4):193–4. 10.1016/S2213-8587(21)00049-8. 10.1016/S2213-8587(21)00049-8 33662332

[3] Thyroid cancer facts and figures. National Cancer Institute Surveillance, Epidemiology, and End Results Program 2021 [Available from: http://seer.cancer.gov/statfacts/html/thyro.html.

[4] Song E, Jeon MJ, Oh H-S, Han M, Lee Y-M, Kim TY, et al. Do aggressive variants of papillary thyroid carcinoma have worse clinical outcome than classic papillary thyroid carcinoma? Eur J Endocrinol. 2018;179(3):135–42. 10.1530/EJE-17-0991. 29875289 10.1530/EJE-17-0991

[5] Agafonoff S, Allamaneni S, Bernstein J, Braverman T, Naqvi I, Chuchulo A. Hypervascular neck mass as the initial presentation of papillary thyroid cancer: a case report and review of current literature. Int J Surg Case Rep. 2020;66:196–200. 10.1016/j.ijscr.2019.12.010. 31865231 10.1016/j.ijscr.2019.12.010 PMC6928285

[6] Aziz NB, Mahmudunnabi RG, Umer M, Sharma S, Rashid MA, Alhamhoom Y, et al. MicroRNAs in ovarian cancer and recent advances in the development of microRNA-based biosensors. Analyst. 2020;145:2038–57. 10.1039/c9an02263e 32016203

[7] Cai H, Hou X, Ding Y, Fu Z, Wang L, Du Y. Prediction of gastric cancer prognosis in the next-generation sequencing era. Tradit Med Mod Med.2019;2(3):105–18.

[8] Srivastava A, Creek DJ. Discovery and validation of clinical biomarkers of cancer: a review combining metabolomics and proteomics. Proteomics. 2019;19(10):1700448. 10.1002/pmic.201700448. 10.1002/pmic.201700448 30353665

[9] Zaballos MA, Santisteban P. Key signaling pathways in thyroid cancer. J Endocrinol. 2017;235(2):R43–r61. 10.1530/JOE-17-0266. 28838947 10.1530/JOE-17-0266

[10] Sharov AA, Schlessinger D, Ko MS. ExAtlas: an interactive online tool for meta-analysis of gene expression data. J Bioinforma Comput Biol. 2015;13(06):1550019. 10.1142/S0219720015500195. 10.1142/S0219720015500195 PMC5518779 26223199

[11] Hong N, Zhang N, Wu H, Lu S, Yu Y, Hou L, et al. Preliminary exploration of survival analysis using the OHDSI common data model: a case study of intrahepatic cholangiocarcinoma. BMC Med Inform Decis Mak. 2018;18(5):81–8. 30526572 10.1186/s12911-018-0686-7 PMC6284277

[12] Goel MK, Khanna P, Kishore J. Understanding survival analysis: Kaplan-Meier estimate. Int J Ayurveda Res. 2010;1(4):274–8. 10.4103/0974-7788.76794. 21455458 10.4103/0974-7788.76794 PMC3059453

[13] Tang Z, Kang B, Li C, Chen T, Zhang Z. GEPIA2: an enhanced web server for large-scale expression profiling and interactive analysis. Nucleic Acids Res. 2019;47(W1):W556–W60. 10.1093/nar/gkz430. 31114875 10.1093/nar/gkz430 PMC6602440

[14] de Hoon MJ, Imoto S, Nolan J, Miyano S. Open source clustering software. Bioinformatics. 2004;20(9):1453–4. 10.1093/bioinformatics/bth078. 14871861 10.1093/bioinformatics/bth078

[15] Ito Y, Miyauchi A, Kihara M, Fukushima M, Higashiyama T, Miya A. Overall survival of papillary thyroid carcinoma patients: a single-institution long-term follow-up of 5897 patients. World J Surg. 2018;42(3):615–22. 10.1007/s00268-018-4479-z. 29349484 10.1007/s00268-018-4479-z PMC5801380

[16] Franceschi S, Lessi F, Panebianco F, Tantillo E, La Ferla M, Menicagli M, et al. Loss of c-KIT expression in thyroid cancer cells. PLoS One. 2017;12(3):e0173913. 10.1371/journal.pone.0173913 PMC5354407 28301608

[17] Robbins HL, Hague A. The PI3K/Akt pathway in tumors of endocrine tissues. Front Endocrinol. 2016;6:188. 10.3389/fendo.2015.00188 PMC4707207 26793165

[18] Kim M-J, Kim SK, Park HJ, Chung DH, Park H-K, Lee JS, et al. PDGFRA promoter polymorphisms are associated with the risk of papillary thyroid cancer. Mol Med Rep. 2012;5(5):1267–70. 10.3892/mmr.2012.784. 22327316 10.3892/mmr.2012.784

[19] Zhang J, Wang P, Dykstra M, Gelebart P, Williams D, Ingham R, et al. Platelet-derived growth factor receptor-α promotes lymphatic metastases in papillary thyroid cancer. J Pathol. 2012;228(2):241–50. 10.1002/path.4069. 22744707 10.1002/path.4069

[20] Vella V, Malaguarnera R. The emerging role of insulin receptor isoforms in thyroid cancer: clinical implications and new perspectives. Int J Mol Sci. 2018;19(12):3814. 10.3390/ijms19123814. 30513575 10.3390/ijms19123814 PMC6321330

[21] Qu T, Li YP, Li XH, Chen Y. Identification of potential biomarkers and drugs for papillary thyroid cancer based on gene expression profile analysis. Mol Med Rep. 2016;14(6):5041–8. 10.3892/mmr.2016.5855. 27779685 10.3892/mmr.2016.5855 PMC5355717

[22] Mitsiades CS, Hayden P, Kotoula V, McMillin DW, McMullan C, Negri J, et al. Bcl-2 overexpression in thyroid carcinoma cells increases sensitivity to Bcl-2 homology 3 domain inhibition. J Clin Endocrinol Metab. 2007;92(12):4845–52. 10.1210/jc.2007-0942. 17848408 10.1210/jc.2007-0942

[23] Tan J, Qian X, Song B, An X, Cai T, Zuo Z, et al. Integrated bioinformatics analysis reveals that the expression of cathepsin S is associated with lymph node metastasis and poor prognosis in papillary thyroid cancer. Oncol Rep. 2018;40(1):111–22. 10.3892/or.2018.6428. 29749483 10.3892/or.2018.6428 PMC6059735

[24] Shin E, Koo JS. Expression of proteins related to autotaxin–lysophosphatidate signaling in thyroid tumors. J Transl Med. 2019;17(1):288. 10.1186/s12967-019-2028-7. 31455351 10.1186/s12967-019-2028-7 PMC6712878

[25] Cong D, He M, Chen S, Liu X, Liu X, Sun H. Expression profiles of pivotal microRNAs and targets in thyroid papillary carcinoma: an analysis of The Cancer Genome Atlas. OncoTargets Ther. 2015;8:2271. 10.2147/OTT.S85753 PMC4556042 26345235

[26] Shang J, Ding Q, Yuan S, Liu J-X, Li F, Zhang H. Network analyses of integrated differentially expressed genes in papillary thyroid carcinoma to identify characteristic genes. Genes. 2019;10(1):45. 10.3390/genes10010045. 30646607 10.3390/genes10010045 PMC6356810

[27] Redler A, Di Rocco G, Giannotti D, Frezzotti F, Bernieri MG, Ceccarelli S, et al. Fibroblast growth factor receptor-2 expression in thyroid tumor progression: potential diagnostic application. PLoS One. 2013;8(8):e72224. 10.1371/journal.pone.0072224 PMC3747152 23977259

[28] Kasaian K, Wiseman SM, Walker BA, Schein JE, Zhao Y, Hirst M, et al. The genomic and transcriptomic landscape of anaplastic thyroid cancer: implications for therapy. BMC Cancer. 2015;15(1):984. 10.1186/s12885-015-1955-9. 26680454 10.1186/s12885-015-1955-9 PMC4683857

[29] Jhunjhunwala S, Jiang Z, Stawiski EW, Gnad F, Liu J, Mayba O, et al. Diverse modes of genomic alteration in hepatocellular carcinoma. Genome Biol. 2014;15(8):436. 10.1186/s13059-014-0436-9. 25159915 10.1186/s13059-014-0436-9 PMC4189592

[30] Zhang Y, Li F, Chen J. MYC promotes the development of papillary thyroid carcinoma by inhibiting the expression of lncRNA PAX8-AS1: 28. Oncol Rep. 2019;41(4):2511–7. 10.3892/or.2019.6996. 30720110 10.3892/or.2019.6996

[31] Huang W, Gu J, Tao T, Zhang J, Wang H, Fan Y. MiR-24-3p inhibits the progression of pancreatic ductal adenocarcinoma through LAMB3 downregulation. Front Oncol. 2020;9:1499. 10.3389/fonc.2019.01499. 10.3389/fonc.2019.01499 PMC6985431 32039003

[32] Han J, Chen M, Wang Y, Gong B, Zhuang T, Liang L, et al. Identification of biomarkers based on differentially expressed genes in papillary thyroid carcinoma. Sci Rep. 2018;8(1):1–11. 29967488 10.1038/s41598-018-28299-9 PMC6028435

[33] Hosseinkhan N, Honardoost M, Blighe K, Moore C, Khamseh M. Comprehensive transcriptomic analysis of papillary thyroid cancer: potential biomarkers associated with tumor progression. J Endocrinol Investig. 2020;43(7):911–23. 10.1007/s40618-019-01175-7 31965517

[34] Qiu J, Zhang W, Xia Q, Liu F, Zhao S, Zhang K, et al. Investigating the mechanisms of papillary thyroid carcinoma using transcriptome analysis. Mol Med Rep. 2017;16(5):5954–64. 10.3892/mmr.2017.7346. 28849102 10.3892/mmr.2017.7346 PMC5865774

[35] Schulten H-J, Alotibi R, Al-Ahmadi A, Ata M, Karim S, Huwait E, et al. Effect of BRAF mutational status on expression profiles in conventional papillary thyroid carcinomas. BMC Genomics. 2015;16(S1):S6. 10.1186/1471-2164-16-S1-S6. 25922907 10.1186/1471-2164-16-S1-S6 PMC4315163

[36] Kato S, Kobayashi T, Yamada K, Nishii K, Sawada H, Ishiguro H, et al. Expression of erbB receptors mRNA in thyroid tissues. Biochim Biophys Acta. 2004;1673(3):194–200. 15279891 10.1016/j.bbagen.2004.04.016

[37] Sun J, Shi R, Zhao S, Li X, Lu S, Bu H, et al. E2F8, a direct target of miR-144, promotes papillary thyroid cancer progression via regulating cell cycle. J Exp Clin Cancer Res. 2017;36(1):40. 10.1186/s13046-017-0504-6. 28270228 10.1186/s13046-017-0504-6 PMC5341194

[38] Kim MK, Park SW, Kim SK, Park HJ, Eun YG, Kwon KH, et al. Association of Toll-like receptor 2 polymorphisms with papillary thyroid cancer and clinicopathologic features in a Korean population. J Korean Med Sci. 2012;27(11):1333–8. 10.3346/jkms.2012.27.11.1333. 23166414 10.3346/jkms.2012.27.11.1333 PMC3492667

[39] Leone V, D’Angelo D, Rubio I, de Freitas PM, Federico A, Colamaio M, et al. MiR-1 is a tumor suppressor in thyroid carcinogenesis targeting CCND2, CXCR4, and SDF-1α. J Clin Endocrinol Metab. 2011;96(9):E1388–E98. 10.1210/jc.2011-0345. 21752897 10.1210/jc.2011-0345

[40] Zhu W, Li C, Ai Z. Candidate agents for papillary thyroid cancer identified by gene expression analysis. Pathol Oncol Res. 2013;19(3):597–604. 10.1007/s12253-013-9625-1. 23519608 10.1007/s12253-013-9625-1

[41] Yamashita AS, Geraldo MV, Fuziwara CS, Kulcsar MAV, Friguglietti CUM, da Costa RB, et al. Notch pathway is activated by MAPK signaling and influences papillary thyroid cancer proliferation. Transl Oncol. 2013;6(2):197. 10.1593/tlo.12442. 23544172 10.1593/tlo.12442 PMC3610552

[42] Lee K-Y, Huang SM, Li S, Kim J-M. Identification of differentially expressed genes in papillary thyroid cancers. Yonsei Med J. 2009;50(1):60–7. 10.3349/ymj.2009.50.1.60. 19259350 10.3349/ymj.2009.50.1.60 PMC2649849

[43] Liang W, Sun F. Identification of key genes of papillary thyroid cancer using integrated bioinformatics analysis. J Endocrinol Investig. 2018;41(10):1237–45. 10.1007/s40618-018-0859-3. 29520684 10.1007/s40618-018-0859-3

[44] Lee EJ, Gusev Y, Jiang J, Nuovo GJ, Lerner MR, Frankel WL, et al. Expression profiling identifies microRNA signature in pancreatic cancer. Int J Cancer. 2007;120(5):1046–54. 10.1002/ijc.22394. 17149698 10.1002/ijc.22394 PMC2680248

[45] Lv Y, Huang S. Role of non-coding RNA in pancreatic cancer. Oncol Lett. 2019;18(4):3963–73. 10.3892/ol.2019.10758. 31579086 10.3892/ol.2019.10758 PMC6757267

[46] Boedigheimer MJ, Wolfinger RD, Bass MB, Bushel PR, Chou JW, Cooper M, et al. Sources of variation in baseline gene expression levels from toxicogenomics study control animals across multiple laboratories. BMC Genomics. 2008;9(1):285. 10.1186/1471-2164-9-285. 18549499 10.1186/1471-2164-9-285 PMC2453529

[47] Shinohara M, Chung YJ, Saji M, Ringel MD. AKT in thyroid tumorigenesis and progression. Endocrinology. 2007;148(3):942–7. 10.1210/en.2006-0937. 16946008 10.1210/en.2006-0937

[48] Xu Y, Han Y-F, Zhu S-J, Dong J-D, Ye B. miRNA-148a inhibits cell growth of papillary thyroid cancer through STAT3 and PI3K/AKT signaling pathways. Oncol Rep. 2017;38(5):3085–93. 10.3892/or.2017.5947. 28901486 10.3892/or.2017.5947

